# Radiological, Clinical, and Surgical Signs of Breast Cancer in Young Women at HLUHS KC

**DOI:** 10.3390/medicina61081429

**Published:** 2025-08-08

**Authors:** Monika Škimelytė, Paula Sungailė, Lukas Dambrauskas, Paulina Paškevičiūtė, Mantas Šližinskas

**Affiliations:** 1 Department of Radiology, Medical Academy, Faculty of Medicine, Lithuanian University of Health Sciences, LT-50161 Kaunas, Lithuania; monika.skimelyte@kaunoklinikos.lt (M.Š.); lu.dambrauskas@gmail.com (L.D.); paulepaskeviciute@gmail.com (P.P.); slizinskas.m@gmail.com (M.Š.); 2 Department of Surgery, Medical Academy, Faculty of Medicine, Lithuanian University of Health Sciences, LT-50161 Kaunas, Lithuania

**Keywords:** breast cancer, young women, radiological examinations

## Abstract

*Background and Objectives*: Breast cancer is the most common malignant disease among women. The aim of this study is to compare clinical, histological, and radiological findings of breast cancer between younger and older women. *Materials and Methods*: This retrospective study enrolled 241 patients with histologically diagnosed breast cancer from January 2015 to December 2021. The patients were divided into two groups: the first group comprised young women aged 49 years or younger and the second group consisted of older women aged over 70 years. Because preventive mammograms were only performed on patients between the ages of 50 and 69 until 2025, we did not include this interval in the study; therefore, only younger and older women who had not undergone preventive mammograms were selected. All patients underwent radiological examinations, including mammography, ultrasound, and magnetic resonance imaging. The parameters were compared between the two groups to evaluate clinical, histological, and radiological features. *Results*: During the study period, a total of 241 patients were included in the final analysis, with 94 (39%) being younger women and 147 (61%) being older women. Clinical signs were analyzed, revealing that redness and swelling (19%) and pain (17.7%) were statistically significantly more common in the older women group compared to the younger group (*p* < 0.013 and *p* < 0.002, respectively). The hormone receptor status of patients in both cohorts did not differ significantly, except for human epidermal growth factor receptor 2 (HER2). Older patients had a significantly higher percentage of HER2-negative disease (83.7%) compared to younger patients (70.2%) (*p* < 0.013). Older women were statistically significantly more likely to have G2 (68.5%) and G3 (21.7%) tumors compared to younger women (G2—36.6% and G3—46.6%) (*p* < 0.001). Ki67 < 40% (61.2%) was statistically significantly more common in the older women group, while Ki67 ≥ 40% was more prevalent in the younger women group (*p* < 0.001). Lobular (19.7%) and ductal (62.6%) histological types of cancer were more common in the older women group (*p* < 0.001). Comparing cancer changes in MRI and ultrasound scans with the results of postoperative histology showed a sensitivity of 87.8% for MRI and 82.7% for ultrasound. Our study suggests that younger women have a higher percentage of proliferation index Ki67 > 40% (73.9%), which is statistically more significant than in the older women group with Ki67 < 40% (63%) (*p* < 0.001). *Conclusions*: All diagnostic tools are essential for early breast cancer detection. Malignant microcalcifications are typically identified through mammography. Breast MRI was found to be more sensitive in detecting breast cancer compared to mammography and ultrasound. While ultrasound is considered the most sensitive and specific diagnostic tool for axillary lymph node evaluation, it is unfortunately not sensitive enough to determine the exact extent of cancer spread. During our retrospective study, T1 and T2 histological sizes were identified most frequently; the earlier the diagnosis is made, the higher the chances of survival and improved quality of life.

## 1. Introduction

Breast cancer is the most common form of cancer and a leading cause of death among women globally. According to the World Cancer Research Fund, breast cancer was the most prevalent cancer in women worldwide, with 2.3 million new cases accounting for 25.8% of all cancer diagnoses in 2020 [1]. Additionally, the Lithuanian National Cancer Center reported that breast cancer was the leading cause of death for women in 2015 [2]. This global disease, which affects millions of women, necessitates effective prevention strategies and accurate diagnoses for early treatment.

Determining the histological subtype of breast cancer is crucial for optimizing treatment outcomes, making biopsy the gold standard procedure. Given the invasive nature of biopsies, there is a continuous effort to identify optimal non-invasive imaging diagnostic tools. Mammography is a widely used screening technique for breast cancer detection that has been proven to effectively reduce mortality rates. However, mammography’s sensitivity is diminished in women with dense breast tissue [3]. Other radiological screening methods include ultrasound, which is commonly used in conjunction with mammography to enhance the visualization of dense breast tissue and lymph nodes (LNs) [4]. Furthermore, magnetic resonance imaging (MRI) is gaining popularity due to its increased sensitivity in cancer detection [5].

Clinical Symptoms: The dominant signs of breast cancer in women include breast pain, nipple discharge, deformation of the breast and/or nipple, palpable lumps, and enlarged lymph nodes. Additional symptoms may involve weight loss, bone pain, and fatigue [6].

Risk Factors: The risk of developing breast cancer increases with age; according to the World Health Organization, it predominantly affects women over the age of 40 [7]. The second largest risk factor is family history. A first-degree family history of breast cancer doubles the likelihood of developing the disease [8]. Women with genetic mutations in BRCA-1 and BRCA-2 have an 80% higher chance of developing breast and ovarian cancers. Other contributing factors include elevated body mass index (BMI), alcohol and tobacco use, and early menarche [8].

Breast cancer in young women has garnered considerable interest, as younger women are often excluded from screening programs. Few studies have focused on the specific symptoms of breast cancer in this demographic. The aim of this study is to retrospectively evaluate the clinical, histological, and radiological features of breast cancer in young and older women. Additionally, it seeks to assess the primary radiological signs of breast cancer across different imaging modalities to determine the most sensitive methods for cancer detection.

## 2. Materials and Methods

Criteria for Patient Selection and Search: This retrospective study involved female patients with histologically diagnosed breast cancer. Patients were included from January 2015 to December 2022. A total of 241 patients were enrolled in this study and were divided into two groups: the first group consisted of young women aged 49 years or younger, while the second group included older women aged 70 years or older. Because preventive mammograms were only performed on patients between the ages of 50 and 69 until 2025, we did not include this interval in the study; therefore, only younger and older women who had not undergone preventive mammograms were selected.

All patients underwent radiological imaging procedures, including mammography, ultrasound, and magnetic resonance imaging, at the HLUHS Department of Radiology.

Statistical Analysis: SPSS (IBM SPSS Statistics 30.0, Statistical Package for the Social Sciences) and Microsoft 365^®^ were used to perform data transformations, collapse categories, and analyze differences. Patient data and characteristics were expressed using descriptive statistics, with absolute (*n*) and percentage frequencies (%) used to assess the distribution of the characteristics in the selected sample. Quantitative data were presented as arithmetic means (m) with standard deviations (SD), medians (Md), and minimum (min) and maximum (max) values. The distributions of variables from two dependent samples were compared using the Wilcoxon test. Differences in patient characteristics were analyzed using appropriate test statistics, including the chi-squared test for categorical data, and pairwise comparisons were performed using the Z-test with the Bonferroni method. When subgroup frequencies were less than five, Fisher’s exact test was applied. The comparison of binary variables for more than two dependent samples was conducted using Cochran’s Q test. The dependence of one binary dependent variable (Y) on several independent variables (X) was evaluated using multivariable logistic regression, with the likelihood ratio (GS) calculated along with its 95% confidence interval (CI). A *p*-value of less than 0.05 was considered statistically significant, and 95% confidence intervals were reported where applicable. The results are presented in charts and tables to display the collected data.

## 3. Results

During the study period, a total of 241 patients were included in the final analysis, consisting of 94 (39%) younger women and 147 (61%) older women (Table 1). The youngest participant was 22 years old, the oldest was 89 years old, and the mean age was 62.04 years. The clinical signs were analyzed, revealing that redness and swelling (19%) and pain (17.7%) were statistically significantly more common in the older women group compared to the younger group (*p* < 0.013 and *p* < 0.002, respectively). The least common clinical signs were discharge from the nipple (3.3%) and inverted nipple (5.4%). During the clinical examination, a lump in the breast was found in 191 patients (79.3%), and lymph nodes in the armpit were identified in 38 patients (15.8%).

Histological features of breast cancer were also analyzed. The hormone receptor status of patients in both cohorts did not differ significantly, except for the human epidermal growth factor receptor 2 (HER2). Older patients had a significantly higher percentage of HER2-negative disease (83.7%) compared to younger patients (70.2%) (*p* < 0.013). Older women were statistically significantly more likely to have G2 (68.5%) and G3 (21.7%) tumors compared to younger women (G2—36.6% and G3—46.6%) (*p* < 0.001). The spread of cancer in lymph vessels (L), blood vessels (V), and resectability (R) during surgery did not differ statistically significantly between the two groups. Ki67 < 40% was statistically significantly more common in the older women group (61.2%), while Ki67 ≥ 40% was more prevalent in the younger women group (*p* < 0.001). Lobular (19.7%) and ductal (62.6%) histological types of cancer were more common in the older women group (*p* < 0.001). The most common type of cancer in the younger women group was ductal carcinoma (92.6%), although this difference was not statistically significant. The histological size of the cancer (pT) did not differ significantly between the two groups. The study found that Stage T1 (56%) and Stage T2 (34.9%) cancers were the most common.

Comparisons of cancer changes in MRI and ultrasound scans with postoperative histology results showed sensitivities of 87.8% for MRI and 82.7% for ultrasound. The sensitivity of these scans increased significantly (*p* < 0.001) after chemotherapy, suggesting a reduction in infiltration in the surrounding tissue of cancer. Due to the predominance of dense glandular tissue, the sensitivity of mammography was only 28.9%. However, after chemotherapy, the accuracy of cancer measurement in mammography improved significantly (*p* < 0.001). Among all radiological examinations, the cancer changes measured during the MRI scans were more often consistent with histological results. According to the study [9], MRI allows for a more accurate determination of the size of the tumor, its multicentricity, and possible changes in the other breast. Treatment tactics are determined based on MRI, which aids in reducing the number of mastectomies.

To evaluate the usefulness of MRI and ultrasound in detecting histological results, it was found that ultrasound was more sensitive (56.3%) than MRI (48.4%), while MRI had higher specificity (82.5%) compared to ultrasound (77.6%).

**Figure 1:**
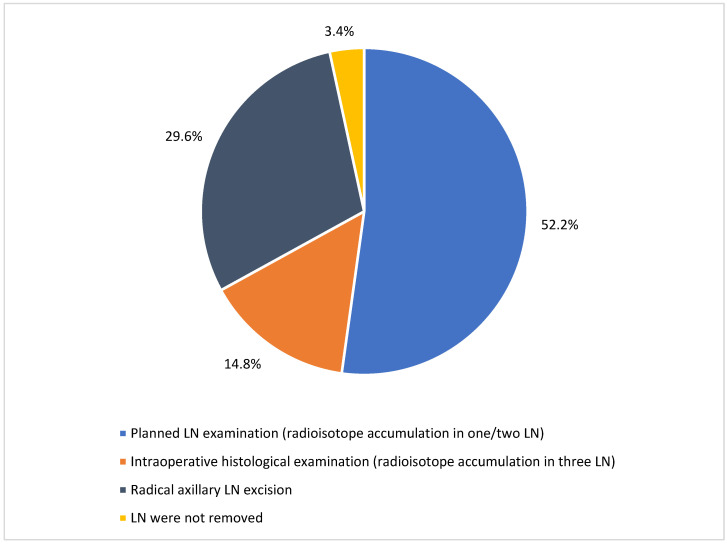
Extent of regional lymph node removal.

Of the 203 patients who underwent surgical treatment, 143 (70.1%) had a lumpectomy and 60 (29.9%) underwent a mastectomy. Out of these 203 cases, 196 (96.6%) women underwent lymph node removal. Most lymph nodes were removed due to radioisotope accumulation in one or two lymph nodes (106 cases; 52.2%), while radical axillary lymph node excision was performed in 60 cases (29.6%). In 30 cases (14.8%), three lymph nodes were removed due to radioisotope accumulation and were sent for intraoperative histological examination. Lymph nodes were not removed in seven patients (3.4%) because pathological lymph nodes were not palpated or detected by radiological examinations (Figure 1).

**Figure 2:**
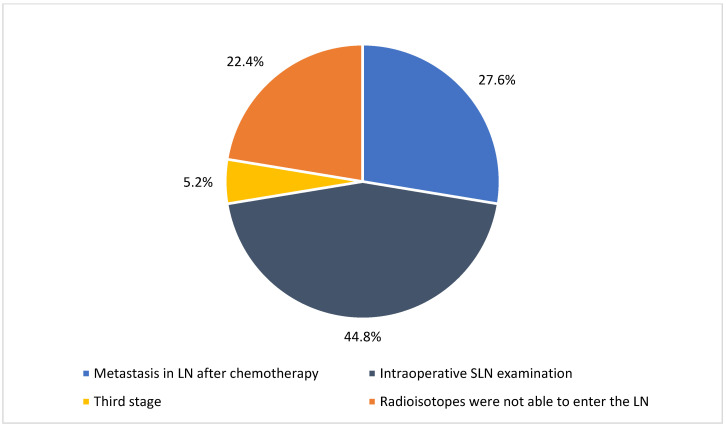
Indications for regional lymph node removal.

Notably, total axillary lymph node excision was performed when metastasis was confirmed in the sentinel lymph node (SLN) in 44.8% of cases. Lymphadenectomy was performed frequently, as radioisotopes were unable to enter the lymph nodes (22.4%) and remained pathological (27.6%) after chemotherapy (Figure 2).

Comparisons were made between age groups and genetic predisposition using Z-tests for continuous variables and the chi-squared test for categorical variables. Notably, the BRCA1 gene mutation was more common in the younger women group (*p* < 0.001).

Of the 241 cases, distant spread of the tumor was detected in 29 cases (12.03%), with no statistically significant difference between the age groups. The most common sites for metastasis included the bones, liver, lungs, and brain, with other notable cases involving the opposite breast, pleura, mediastinum, or neck lymph nodes.

One of the objectives of this study was to identify significant radiological signs in tumors. In the ultrasound analysis, there were statistically significant findings of hypovascular (71.4%; *p* < 0.001) and hypoechoic (76.2%; *p* < 0.025) tumors in the older women group, with no diffuse infiltration of tumor lesions observed (74%; *p* < 0.041).

No statistically significant radiological evidence was observed in the mammography (MG) analysis, as tumor lesions were not sufficiently differentiated due to dense glandular tissue. In the MRI analysis, tumor lesions exhibited significantly greater accumulation in the older group (97.3%; *p* < 0.044) compared to the younger group (91.5%).

Among the 241 patients, 90 cases (37.3%) of multifocal tumors were detected, along with 80 cases (33.2%) featuring pathological blood vessels and suspected pathological lymph nodes in 75 cases (31.1%). The Ki67 proliferation index (greater than 40%) was significantly higher in the older women group, while HER2 receptors were negative (*p* < 0.036) and estrogen status was positive (*p* < 0.03).

Our study suggests that younger women have a higher percentage of a Ki67 proliferation index greater than 40% (73.9%), which is statistically more significant than in the older women group with a Ki67 index less than 40% (63%) (*p* < 0.001).

Out of 241 cases, 77 (32%) were found to be TNBC and 38 cases (15.8%) were found to be TPBC. Regarding TPBC, there was a statistically significant difference between the patient groups, with younger women being more likely to have TPBC (55.3%; *p* < 0.023) compared to older women. In the younger women group, we found predominant prognostic parameters such as a higher degree of differentiation (G2—47.4%; G3—42.1%) and a statistically significantly higher receptor and cell proliferation index (Ki67 > 40–69.4%; *p* < 0.022), along with smaller tumor size and earlier stage (T1—60.5%; T2—31.6%).

TNBC tumors were more commonly found in the older women group (59.7%) with a greater degree of differentiation (G2—50%; G3—39.7%). Typically, these tumors were identified as smaller in size and stage (T1—45.5%, T2—42.9%) with a higher receptor and cell proliferation index (Ki67 > 40–56.9%). However, no statistically significant dependence was found in the calculations.

**Figure 3:**
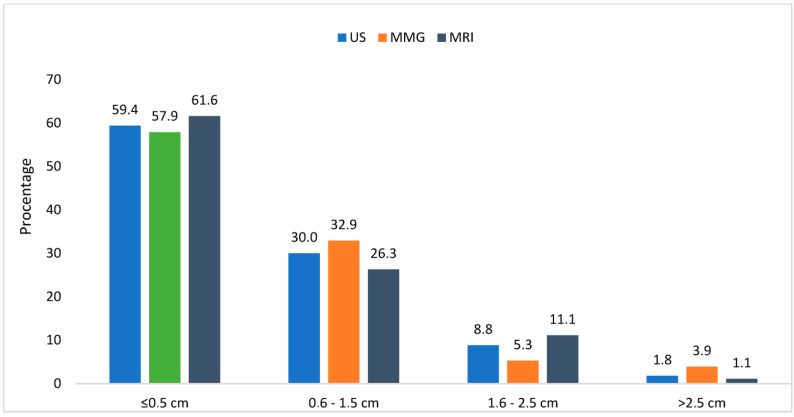
Sensitivity of radiological tests depending on tumor size.

One of our tasks was to evaluate the preoperative radiological examinations of the breasts at HLUHS KC in conjunction with the data from the postoperative pathological histology examinations. An assessment of the structures with an error margin of 0–0.5 cm was considered accurate. The error ranges were divided into the intervals of 0–0.5 cm, 0.6–1.5 cm, 1.6–2.5 cm, and >2.5 cm. From the presented data, we found that HLUHS KC radiologists accurately portrayed tumor sizes (by MG, US, or MRI) with a precision of 58–62%. Errors in hypodiagnostic and hyperdiagnostic measurements were distributed as follows: 0.6–1.5 cm occurring in 26–33% of cases; 1.6–2.5 cm in 5–11%; and >2.5 cm in 1–4%. We can conclude that the minimum radiological error in the assessment of tumor sizes starts from 0 to 1.5 cm (inclusive). Our findings suggest that the sensitivity of the radiologist as an evaluator is between 88 and 91% across all radiological tests (US, MG, and MRI) (Figure 3).

## 4. Discussion

In our retrospective cohort study, we aimed to determine the most common radiological signs of breast cancer in young women, compare the diagnostic methods employed, and assess the results in relation to those of older women. Based on our findings, the ultrasound examinations indicated that the older women exhibited significantly higher signs of hypovascularity (*p* < 0.001), hypoechoic tumors (*p* < 0.025), and diffuse infiltration (*p* < 0.041) compared to younger women. Additionally, triple-positive (TPBC) breast cancer (*p* < 0.023) and a Ki67 proliferative index of ≥40% (*p* < 0.022) were significantly more prevalent among younger women.

Prior studies have also demonstrated that TPBC breast cancer is more common in younger women. Breast cancer occurring at a younger age tends to exhibit more aggressive characteristics, higher tumor grades, and increased proliferation fractions, along with greater vascular invasion compared to similar malignancies found in older women. Furthermore, younger women face numerous psychological challenges, including a higher incidence of radical mastectomy, premature menopause, and infertility. Various studies have identified young age as an independent factor associated with poor disease prognosis. Ivan Erić et al. [10] found that early-onset breast carcinoma had a higher frequency of tumor grade 3 (29% vs. 17%) and estrogen receptor negativity (45% vs. 23%). In the group of young women, breast carcinoma was predominantly multicentric (23% vs. 5%), as well as triple-negative (TNBC) (32% vs. 10%), and exhibited a higher Ki67 proliferation index (25% vs. 10%). These results confirm that younger women are more likely to have aggressive forms of cancer compared to older women. According to Jennifer L. et al. [11] younger women were more likely to present with tumors that were higher grade, larger in size, ER/PR-negative, and lymph node-positive (*p* < 0.001). Moreover, younger women were more likely to die from breast cancer compared to older women (cHR 1.39, CI 1.34–1.45).

In terms of radiological screening methods, we compared mammography, ultrasound, and MRI. Most of the young patients had dense breast tissue, which lowers detection sensitivity. As a result of this dense tissue and larger breast size, mammography exhibited a sensitivity of only 28.9%. Despite this low sensitivity, mammography is still considered the gold standard for the early detection of breast cancer. The most precise imaging method was found to be MRI, with a sensitivity of 48.4% and a specificity of 82.5%. Our findings align with those of Eun L. Langman et al. [12] who noted that women undergoing additional MRI examinations were found to have more suspicious findings that were not detected by mammograms or ultrasounds. MRI plays a significant role in further clinical treatment, and accurate imaging methods are essential for any surgical team. Thus, MRI is an excellent radiological tool for assessing large and dense breasts in young women.

Ultrasound also plays a critical role in the radiological examination of breasts. Our study found that ultrasound was more sensitive (56.3%) than MRI (48.4%), although MRI had higher specificity (82.5%) compared to ultrasound (77.6%). Breast ultrasound is recommended as a supplementary method to mammography, particularly for patients at high risk of breast cancer, for the detection of malignant lymph nodes, and for the additional examination of dense breast tissue.

## 5. Conclusions

The main focus of this study was to assess the pathomorphological and radiological features of primary breast cancer in women of different age groups and to explore the correlation between preoperative radiological examination data and the pathological histological findings obtained after surgery. It is important to acknowledge that our study has limitations; specifically, the retrospective nature of the research and the collection of data from a single institution may restrict the generalizability of our findings.

Our analysis revealed that breast cancer in younger women is more often multicentric, diffusely spreading, and characterized by high-grade differentiation, TPBC status, and a higher proliferation index (Ki67). These findings support the hypothesis that young women generally exhibit a more aggressive form of cancer compared to older women. Local redness, swelling, and soreness of the affected breast were statistically significantly more common in the older women group, while these symptoms were rarely observed in the younger women group. Our results further suggest that more aggressive tumor characteristics necessitate more aggressive treatments, which in turn may influence the survival rates of patients.

**Figure 4:**
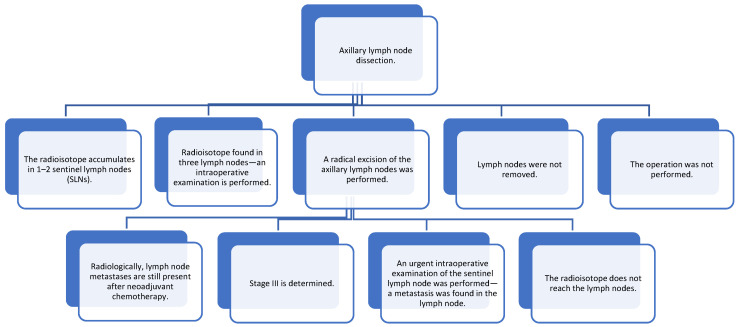
Axillary lymph node dissection.

The use of various diagnostic tools is essential for effective breast cancer detection. Mammography is commonly used to differentiate malignant microcalcifications. Our study found that breast MRI is more sensitive in detecting breast cancer compared to mammography and ultrasound. Furthermore, we observed lower detection rates of breast cancer using ultrasound compared to mammography and MRI. During ultrasound examinations, the extent of cancer spread was considerably smaller compared to that assessed using other radiological tools. Given the diffuse and multicentric nature of cancer, MRI is generally more effective for detection. While ultrasound is regarded as the most sensitive and specific diagnostic tool for evaluating axillary lymph nodes (Figure 4), it is crucial to note that earlier diagnosis—particularly of T1 and T2 histological sizes—correlates with a higher likelihood of survival and improved quality of life. Based on the literature reviewed, it is important to educate the public and talk about the signs of breast cancer and possible preventive measures. It is important to remind the public about diagnostic tests that can diagnose cancer as early as possible.

## Figures and Tables

**Table 1:** Histopathology.

Histopathology		Age Groups, *n* (%)		Total, (*n* = 241)	*p*
		≤49 Year (*n* = 94)	≥70 Year (*n* = 147)		
Tumor size (pT)	T1	54 (57.4)	81 (55.1)	135 (56.0)	0.650
	T2	34 (36.2)	50 (34.0)	84 (34.9)	
	T3	3 93.2)	6 (4.1)	9 (3.7)	
	T4	3 (3.2)	10 (6.8)	13 (5.4)	
Tumor type	Ductal	87 (92.6)	92 (62.6)	179 (74.3)	<0.001
	Lobular	0	29 (19.7)	29 (12.0)	
	Papillar	1 (1.1)	7 (4.8)	8 (3.3)	
	In situ	6 (6.4)	18 (12.2)	24 (10)	
ER	Negative	38 (40.9)	54 (36.7)	92 (38.3)	0.522
	Positive	55 (59.1)	93 (63.3)	148 (61.7)	
PR	Negative	46 (49.5)	67 (45.6)	113 (47.1)	0.557
	Positive	47 (50.5)	80 (54.4)	127 (52.9)	
HER2	Negative	66 (70.2)	123 (83.7)	189 (78.4)	0.013
	Positive	28 (29.8)	24 (16.3)	52 (21.6)	
G	G1	5 (5.5)	14 (9.3)	19 (7.9)	<0.001
	G2	34 (37.3)	98 (65.3)	132 (54.8)	
	G3	43 (47.3)	31 (20.7)	74 (30.7)	
	Fibrosis	9 (9.9)	7 (4.7)	16 (6.6)	
Ki67	0	1 (1.1)	4 (2.9)	5 (2.2)	<0.001
	<40%	24 (25.8)	85 (61.2)	109 (47.0)	
	≥40%	68 (73.1)	50 (36.0)	118 (50.9)	

## Data Availability

The original contributions presented in this study are included in the article. Further inquiries can be directed to the corresponding author.
